# Isothiocyanates as Ferroptosis Inducers to Promote Cancer Cell Death

**DOI:** 10.1002/ptr.70389

**Published:** 2026-05-20

**Authors:** Valentina Citi, Renda Chahna, Francesca Maffei, Alma Martelli, Vincenzo Calderone, Carmela Fimognari, Eleonora Turrini

**Affiliations:** ^1^ Department of Pharmacy University of Pisa Pisa Italy; ^2^ Interdepartmental Research Centre Nutraceuticals and Food for Health‐NUTRAFOOD, University of Pisa Pisa Italy; ^3^ Interdepartmental Research Centre of Ageing Biology and Pathology, University of Pisa Pisa Italy; ^4^ Department for Life Quality Studies Alma Mater Studiorum—Università di Bologna Rimini Italy

**Keywords:** anticancer, ferroptosis, isothiocyanate, PEITC, sulforaphane

## Abstract

Cancer remains a leading cause of global mortality, with the therapeutic efficacy of anticancer drugs often hampered by late diagnosis and the development of chemotherapy resistance. Ferroptosis is an iron‐dependent form of regulated non‐apoptotic cell death driven by lipid peroxidation. It has emerged as a potential therapeutic target to overcome drug resistance in cancer. Isothiocyanates (ITCs) from cruciferous vegetables have garnered attention for their multi‐target anticancer properties. In this review, we present the key mechanisms of ferroptosis and critically evaluate current evidence indicating that ITCs can induce ferroptosis through multiple, compound‐specific redox‐ and iron‐dependent mechanisms. We further discuss whether targeting ferroptosis with ITCs may represent a novel strategy to improve anticancer treatment. Overall, ITCs emerge as versatile but tightly regulated modulators of ferroptosis in cancer, whose effective therapeutic exploitation requires further in vivo and clinical studies, biomarker‐guided dose optimization, and rational combination strategies with standard anticancer chemotherapy to translate their preclinical findings into therapeutic benefit.

## 1 Introduction

Cancer still represents one of the leading causes of death worldwide, posing a public health challenge with increasing incidence and mortality. In 2022, the World Health Organization reported approximately 20 million new cancer cases and nearly 10 million cancer‐related deaths (Bray et al. 2024). In the coming decades, the burden is projected to rise substantially due to aging populations, lifestyle‐related risk factors, and environmental exposures (Li et al. 2024). Despite advances in anticancer therapies such as chemotherapy, radiotherapy, and targeted treatments, many cancers remain difficult to treat due to late diagnosis, resistance to chemotherapy, and therapy‐associated toxicity (Leng et al. 2024).

In this context, naturally derived compounds have gained increasing attention for their potential in both cancer prevention and treatment (Asma et al. 2022; Choudhari et al. 2020). Over half of current anticancer drugs are derived from natural sources or are designed based on natural scaffolds. These bioactive compounds often target multiple signaling pathways and can intervene at various stages of tumor development (Hashem et al. 2022). Among them, isothiocyanates (ITCs), a class of sulfur‐containing compounds derived from glucosinolates found mainly in cruciferous vegetables, have shown considerable promise in cancer research (Joković et al. 2025; Labudda et al. 2026). ITCs can modulate a wide range of cellular processes relevant to cancer, including inhibition of phase I enzymes that activate carcinogens, induction of nuclear factor erythroid 2‐related factor 2 (NRF2)‐regulated genes of phase II detoxification enzymes, cell‐cycle regulation, apoptosis induction, and epigenetic modifications (Sundaram et al. 2022) and continue to attract attention for their potential roles in oncology. The ITCs more extensively investigated for their antitumor properties include allyl isothiocyanate (AITC), benzyl isothiocyanate (BITC), phenethyl isothiocyanate (PEITC), and sulforaphane (Sundaram et al. 2022).

Ferroptosis is an iron‐dependent, non‐apoptotic form of regulated cell death characterized by the accumulation of lipid peroxides and reactive oxygen species (ROS). Unlike apoptosis, ferroptosis is not mediated by caspases but instead involves the dysregulation of iron metabolism, glutathione (GSH) depletion, and inhibition of glutathione peroxidase 4 (GPX4). This mechanism of cell death has emerged as a promising target in cancer therapy, particularly in tumors that exhibit resistance to traditional forms of cell death (Lei et al. 2022; Li et al. 2020; Zhou et al. 2024).

In this review, we provide a novel perspective of ITCs and ferroptosis in oncology. We first present the main mechanisms of ferroptosis and its implications in cancer development. The core of our discussion highlights the most recent evidence supporting ITCs as ferroptosis inducers for cancer treatment. Finally, we critically evaluate whether the ability of ITCs to trigger ferroptosis may offer the basis for a novel strategy to improve anticancer treatments, highlighting strengths, limitations, and future perspectives.

## 2 Leading Mechanisms of Ferroptosis

**FIGURE 1:**
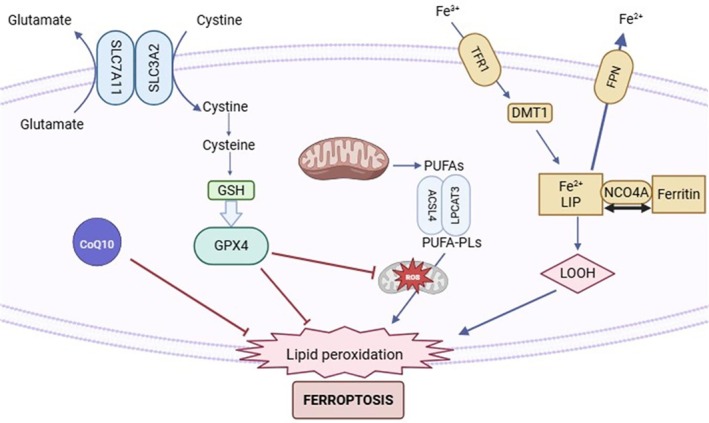
Schematic representation of ferroptotic cell death pathway. ACSL4: Acyl‐CoA synthetase long‐chain family member 4; CoQ10: Coenzyme Q10; DMT1: Divalent metal transporter; FPN: Ferroportin; GSH: Glutathione; GPX4: Glutathione peroxidase 4; LIP: Labile iron pool; LOOH: Lipid hydroperoxides; LPCAT3: Lysophosphatidylcholine acyltransferase 3; NCOA4: Nuclear receptor coactivator 4; PUFAs: Polyunsaturated fatty acids; PUFA‐PLs: Polyunsaturated fatty acid–phospholipids; TFR1: Transferrin receptor 1; Created in BioRender.com.

Ferroptosis is morphologically and mechanistically distinct from the other forms of cell death, such as apoptosis, necroptosis, or pyroptosis. Morphologically, ferroptotic cells exhibit intact nuclei, aberrant mitochondria with reduced cristae and inner membrane condensation, outer membrane rupture, and mitochondrial shrinkage (Liu et al. 2022). The molecular framework underlying ferroptosis integrates three interdependent components: (i) iron metabolism, (ii) lipid peroxidation, (iii) antioxidant defense, whose interplay ultimately dictates ferroptosis sensitivity (Figure 1).

### 2.1 Iron Metabolism

The initiation of ferroptosis is tightly linked to the intracellular accumulation of redox‐active iron within the labile iron pool (LIP). Although iron is essential for mitochondrial energy production and DNA synthesis, an excess of ferrous iron (Fe^2+^) catalyzes ROS formation through Fenton and Haber‐Weiss reactions, generating hydroxyl radicals that trigger lipid peroxidation. Intracellular iron homeostasis relies on the dynamic balance among its uptake, storage, utilization, and export. Ferric iron (Fe^3+^) is internalized through the transferrin (TF)‐transferrin receptor 1 (TFR1) complex and reduced to ferrous (Fe^2+^), while divalent metal transporter (DMT1) mediates direct Fe^2+^ import from the extracellular space at the plasma membrane and from endosomes after transferrin‐mediated iron uptake. Iron in excess is stored in ferritin complexes, consisting of ferritin heavy (FTH1) and light (FTL) chains, which require Fe^2+^ and prevent oxidative damage (Yang and Stockwell 2008). Under stress, nuclear receptor coactivator 4 (NCOA4) mediates ferritinophagy, the selective autophagic degradation of ferritin, releasing catalytic Fe^2+^ that amplifies ferroptosis. Conversely, ferroportin (FPN) exports excess iron, maintaining cytoplasmic balance, and iron homeostasis is regulated by the iron regulatory proteins (IRP) 1 and 2. When intracellular iron is low, IRPs bind the iron response element (IRE) in the 5′‐UTR region of iron metabolism‐related genes to regulate their transcription, inducing for instance gene transcripts of TFR1 and inhibiting FPN (Liu et al. 2022). Dysregulation of these processes, through overexpression of TFR1 or suppression of FPN, increases the LIP and sensitizes cells to ferroptosis (Liu et al. 2022; Wu et al. 2023). Lysosomal iron recycling further reinforces ROS‐mediated oxidative stress. The suppression of ferroptosis by iron chelators such as deferoxamine (DFO) highlights the pivotal role of iron‐dependent ROS generation in the execution of ferroptotic death.

### 2.2 Lipid Peroxidation

At the biochemical level, ferroptosis is defined by the iron‐dependent peroxidation of polyunsaturated fatty acids (PUFAs) in the membrane phospholipids. The susceptibility of cells to ferroptosis correlates directly with their membrane PUFA content (Shah et al. 2018; Yang and Stockwell 2016). Acyl‐CoA synthetase long‐chain family member 4 (ACSL4) catalyzes the conversion of arachidonic acid (AA) and adrenic acid (AdA) into acyl‐CoA derivatives, which are then incorporated into phosphatidylethanolamine (PE) by lysophosphatidylcholine acyltransferase 3 (LPCAT3) to form PE‐AA/AdA complexes. These PUFA‐enriched phospholipids (PUFA‐PLs) are the prime substrates for oxidation by free radicals and lipoxygenases (LOXs), leading to the accumulation of cytotoxic lipid hydroperoxides (PE‐AA/AdA‐OOH) (Kagan et al. 2017; Zhang, Liu, et al. 2022). The two principal mechanisms driving this oxidation are: (i) non‐enzymatic autoxidation, initiated by iron‐catalyzed Fenton reactions; (ii) enzymatic oxidation, mediated by non‐heme iron‐dependent LOXs (Zhang, Liu, et al. 2022). Both pathways yield toxic lipid peroxidation products, such as 4‐hydroxynonenal (4‐HNE) and malondialdehyde (MDA), which compromise membrane integrity and promote cell death. Inhibitors targeting ACSL4, LPCAT3, or LOXs markedly suppress ferroptosis, confirming their essential roles in this death process (Chen et al. 2023).

### 2.3 Antioxidant Defense

The central defense mechanism against ferroptosis is the system of cystine/glutamate antiporter Xc^−^–GSH–GPX4 axis. System Xc^−^, a heterodimer formed by SLC7A11 and SLC3A2, mediates the exchange of extracellular cystine for intracellular glutamate. Imported cystine is reduced to cysteine, which serves as a rate‐limiting precursor for GSH synthesis, catalyzed by glutamate–cysteine ligase (GCLC) (Dixon et al. 2012). GPX4, a selenoenzyme, utilizes GSH to reduce lipid hydroperoxides to non‐toxic lipid alcohols, preserving membrane integrity and redox homeostasis. Depletion of cystine or GSH, therefore, leads to GPX4 inactivation and unrestrained lipid peroxidation (Dixon et al. 2014). Pharmacological agents that exemplify these mechanisms include erastin and RLS3. Erastin inhibits cystine uptake through the Xc^−^system, leading to GSH depletion and indirect suppression of GPX4 activity; RSL3 directly binds and inactivates GPX4 regardless of GSH levels (Liu et al. 2022). Both compounds ultimately disrupt the cellular antioxidant defense system, leading to oxidative imbalance, ROS accumulation, lipid peroxidation, and ferroptosis. The direct or indirect inactivation of GPX4 highlights its role as a key suppressor of ferroptosis, making it a potential therapeutic target in diseases involving ferroptotic cell death (Na et al. 2024). The mevalonate (MVA) pathway is another negative regulator of ferroptosis, contributing to the maintenance of cellular redox homeostasis through the biosynthesis of coenzyme Q10 (CoQ10), which acts as a lipophilic antioxidant preventing lipid peroxidation. Parallel antioxidant mechanisms include the ferroptosis suppressor protein 1 (FSP1)–CoQ10 axis, which regenerates reduced CoQ10 (ubiquinol) in the plasma membrane and provides GPX4‐independent resistance to ferroptosis (Bersuker et al. 2019).

### 2.4 Membrane Damage, Lysosomal Involvement, and Repair Mechanisms

The terminal phase of ferroptosis involves rupture of the plasma membrane and disruption of the mitochondrial outer membrane. Excessive accumulation of ROS beyond the buffering capacity of antioxidant systems results in irreversible membrane damage, culminating in ferroptotic cell death (Jiang et al. 2021). Lipid oxidation also affects lysosomal membranes, whose destabilization releases proteolytic enzymes such as cathepsins, exacerbating cellular destruction (Riegman et al. 2020). To mitigate membrane rupture, the endosomal sorting complex required for transport III (ESCRT‐III) machinery, composed of charged multivesicular body proteins (CHMPs), mediates calcium‐dependent membrane repair. Upon ferroptotic insult, Ca^2+^ influx recruits CHMP5 and CHMP6 to the damaged sites, limiting cell lysis. Loss of ESCRT‐III components increases susceptibility to ferroptosis, underscoring its protective function (Pedrera et al. 2021). Recent studies indicate that ferroptotic cells can release oxidized lipid‐enriched extracellular vesicles, which promote ferroptosis to neighboring cells and amplify tissue damage. The complex interplay among lysosomal activity, membrane repair systems, and antioxidant defenses points out the multifaceted regulation of ferroptosis (Riegman et al. 2020).

## 3 The Role of Ferroptosis in Cancer

Ferroptosis represents an interlinked and highly regulated process integrating iron metabolism, lipid peroxidation, and antioxidant defense. The balance between pro‐oxidant forces and protective mechanisms determines cellular fate. Cancer cells, characterized by elevated iron dependency, ROS generation, and altered lipid metabolism, exhibit specific susceptibility to ferroptosis, which emerges as a promising therapeutic target that can be exploited (Rodriguez et al. 2022; Zhang, Liu, et al. 2022).

Several studies indicate that ferroptosis can act as a tumor suppressor, decreasing cell proliferation, cell cycle, and cancer progression in a variety of solid and hematological cancer models (Chen, Kang, et al. 2021; Chen et al. 2023). Following an initial period of limited recognition, a deeper understanding of ferroptosis's role in cancer and drug resistance has driven intensified research into small molecules capable of inducing this form of cell death. This has led to a rapid increase in the number of published studies on this topic. Drugs such as sulfasalazine or sorafenib, and small molecules such as erastin, RLS3, and buthionine sulfoximine, which work as ferroptosis inducers, have been demonstrated to counteract growth and metastasis of cancers, particularly in iron‐rich tissues, i.e., liver, brain, and pancreas (Zhang, Xin, et al. 2022; Zheng et al. 2021). Erastin and RLS3 are now recognized in pre‐clinical studies as promising anticancer agents for therapy‐resistant cancers, especially where there is apoptosis resistance (Zhang, Liu, et al. 2022). Moreover, key tumor suppressor genes such as p53, BRCA1‐associated protein 1 (BAP1), fumarate hydratase (FH), Kelch‐like ECH‐associated protein 1 (KEAP1), and the epigenetic modulator MLL4 (mixed‐lineage leukemia 4) have been shown to exert their tumor‐suppressive functions, at least partially, by inducing ferroptosis in cancer cells (Zhou et al. 2024). In terms of therapeutic vulnerability, ACSL4 expression serves as a biomarker of ferroptotic sensitivity, with ACSL4‐high tumors displaying greater vulnerability to ferroptosis inducers (Grube et al. 2022).

In cancer cells, ferroptotic cell death can be triggered by targeting NRF2 or interfering with the cysteine/GSH metabolism, and inducing oxidative stress and iron‐mediated cytotoxicity. NRF2 is a master regulator of antioxidant defense, frequently upregulated in various cancers, and acts as a key driver of tumor progression, metastasis, and therapy resistance. Recent evidence suggests that NRF2 may play a dual role in ferroptosis. It counteracts ferroptosis by acting on critical components of the ferroptosis cascade, such as SLC7A11, GPX4, and FSP1, thereby promoting tumor survival and contributing to therapeutic resistance (Zhou et al. 2024). On the other hand, it can trigger ferroptosis by increasing the expression of the protein heme hoxigenase 1 (Hmox1), leading to iron overload and ferroptosis in some tumor cell types (Chen et al. 2023).

Some findings suggest that cancer cells evade ferroptosis to promote tumor growth, survival, and metastasis. There are important connections between tumor suppressor pathways and ferroptosis regulation in cancer progression. Jiang and colleagues revealed that the loss of p53 tumor suppressor function contributes to ferroptosis suppression (Jiang et al. 2015). The study showed that functional p53 restricts cystine import via the cystine/glutamate antiporter Xc^−^, thereby reducing intracellular GSH levels and sensitizing cells to ferroptotic death. When p53 is inactivated, this tumor‐suppressive mechanism is lost, allowing cancer cells to evade ferroptosis.

Ferroptosis may represent a double‐edged sword in human cancer (Lei et al. 2022; Quaranto et al. 2025). Although it is mainly considered a tumor suppressor, one in vivo study indicated that genetic ablation of GPX4 or exposure to a high‐iron diet sped up the development of pancreatic ductal adenocarcinoma induced by kRAS (Dai et al. 2020). Moreover, excessive or poorly targeted induction of ferroptosis may have detrimental consequences, including impairment of immune cell function and promotion of immune evasion within the tumor microenvironment, underscoring the need for precise spatial and temporal modulation of this cell death pathway (Lei et al. 2022; Quaranto et al. 2025). Nevertheless, ferroptosis is typically regarded as a potent tumor‐suppressive mechanism in many tumors and therapeutic settings. Accordingly, numerous studies have focused on the development of ferroptosis inducers for cancer therapy (Chen et al. 2023).

## 4 ITCs as Inducers of Ferroptosis for Cancer Treatment

The overall studies on ITCs as inducers of ferroptosis are reported in Table 1.

**TABLE 1:** In vitro and in vivo ferroptotic anticancer activity of ITCs.

Isothiocyanate	Cancer model	Concentration and time of treatment	IC_50_	Ferroptotic anticancer mechanisms	References
PEITC PEITC in association with cotylenin	Human panceratic cancer cells (MIAPaCa‐2, PANC‐1 and gemcitabine‐resistant PANC‐1 cells)	2–8 μM for 16–24 h PEITC 6 μM and cotylenin 15 μg/mL		Inhibition of cytotoxic activity by ferroptosis inhibitor DFO Inhibition of cytotoxic activity by ferroptosis inhibitors ferrostatin‐1, liproxstatin, and DFO	Kasukabe et al. (2016)
PEITC	Murine osteosarcoma cells (K7M2 cells)	In vitro: 1–15‐30 μM for 4–12–24‐48 h according to experimental exigencies	33.49, 38.23, and 42.06 μM (24, 48, and 72 h, respectively)	In vitro: ↑ total and labile iron ↑ TFR1 ↓ FPN, FTH1 and DMT1 ↑ FTH1 and SLC40A1 mRNAs; ↓ TFRC mRNA ↓ GSH, ↓ GPX4; ↑ lipid peroxidation; ↑ MDA	Lv et al. (2020b)
	Syngeneic orthotopic osteosarcoma mouse model (transplanted with K7M2 into the tibia)	In vivo: 30, 60, 90 mg/kg die for 24 days (intragastrical administration)		In vivo: ↓ GPX4 ↑ TFR1 ↓ FPN and FTH1	
PEITC	Human osteosarcoma cells (MNNG/HOS, U‐2 OS, MG‐63, and 143B cells)	In vitro: 1–15‐30 μM for 4–12–24‐48 h according to experimental exigencies		In vitro: ↑ labile iron ↓ GSH, ↑ ROS generation, ↑ lipid peroxidation, ↑ MDA; ↓ GPX4 ↑ TFR1; ↓ FPN, FTH1, DMT1 and IRP2 ↑ FTH1 and SLC40A1 mRNA; ↓ IRP2, TFRC, and SLC11A2 mRNA	Lv et al. (2020a)
	Xenograft osteosarcoma nude mice (transplanted with MNNG/HOS)	In vivo: 30 mg/kg die for 24 days (intragastrical administration)		In vivo: ↑ TFR1; ↓ FPN and DMT1 ↓ GPX4	
Sulforaphane	Human acute myeloid leukemia cells (U937, MV4‐11 cells)	25 or 50 μM for 24 h		↓ GSH ↓ GPX4 ↑ lipid peroxidation ↑ MDA	Greco et al. (2021)
Sulforaphane	Human small‐cell lung cancer (NCI‐H69, NCI‐H69AR (H69AR) and NCI‐H82 cells)	1–20 μM for 48, 96 h		↑ iron level ↑ lipid peroxidation ↓ SLC7A11 protein and mRNA expression	Iida et al. (2021)
Sulforaphane	Colorectal cancer cells (HCT‐116)	4–16 μM for 12, 24 or 48 h		↑ SIRT3/AMPK/mTOR axis ↑ ROS, MDA, iron level ↓ SLC7A11	Hu et al. (2024)
Sulforaphane	Human Osteosarcoma cells (143B and SJSA‐1)	In vitro: 10 μM for 12 h In vivo: subcutaneous xenograft model 25 or 50 mg/kg every 2 days (intraperitoneal injection) for 18 days Intratibial xenograft model 50 mg/kg every 2 days (intraperitoneal injection) for 16 days		In vitro: Interaction with p62 protein, ↓ SLC7A11 protein expression ↑ ROS generation ↑ lipid peroxidation Morphological mitochondria alternation (↑ membrane density; ↓ or absent cristae) In vivo ↓ tumour volume ↓ GSH, ↑ MDA ↓ Ki67, SLC7A11, and GPX4 ↓ tumour volume ↓ Ki67, SLC7A11, and GPX4	Zou et al. (2025)
Synthetic isothiocyanate containing AR‐antagonists (ITC‐ARis)	Human prostate cancer cells (VCaP, LNCaP, C4–2, 22Rv1, RWPE‐1, and MDA‐kb2 cells)	1–15 μM for 72 h	**3** 4.85 μM **4** 3.89 μM **5** 6.09 μM in VCaP cells	↑ lipid peroxidation ↓ GSH	Qin et al. (2021)

*Note:* ↑ increase; ↓ decrease, degradation.

Abbreviations: AMPK: AMP‐activated protein kinase; DFO: deferoxamine; DMT1: divalent metal transporter; FPN: iron‐efflux protein ferroportin; FTH1: iron storage protein ferritin; GPX4: glutathione peroxidase 4; GSH: glutathione; IC50: half‐maximal inhibitory concentration; IRP2: iron regulatory protein 2; MDA: malondialdehyde; mTOR: mechanistic target of rapamycin; SIRT3: sirtuin3; SLC11A2: solute carrier family 11 member 2; SLC40A1: solute carrier family 40 member 1; TFR1: iron‐uptake protein transferrin receptor‐1; TFRC: transferrin receptor.

### 4.1 PEITC

**FIGURE 2:**
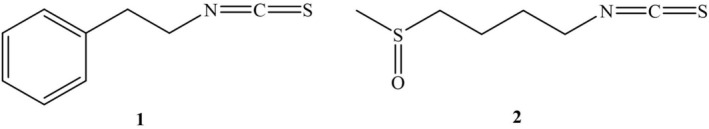
Chemical structure of PEITC (**1**) and sulforaphane (**2**).

The first evidence of ITCs' ability to trigger cancer cell death via ferroptosis was published for PEITC (Figure 2, **1**) in combination with cotylenin, a plant fucicoccan‐diterpene glycoside, in PANC‐1 pancreatic cancer cells (Table 1). The anticancer effects of PEITC strongly depend on its capability to generate ROS. In association with cotylenin, this ability synergistically increased, inducing cell death at concentrations at which neither cotylenin nor PEITC alone affected cell viability (Kasukabe et al. 2016). The use of antioxidants (N‐acetyl‐cysteine (NAC) and trolox) and the ferroptosis inhibitors ferrostatin‐1, liproxstatin, and the lysosomal iron chelator DFO significantly decreased the cytotoxic activity of the combination PEITC—cotylenin (Kasukabe et al. 2016). Apoptosis and necroptosis inhibitors failed to contrast cell death induced by the co‐treatment, and the autophagy inhibitor only partially rescued cell viability, supporting the hypothesis that ferroptosis was the main mechanism evoked by PEITC in combination with cotylenin. Interestingly, ferrostatin‐1 did not inhibit cell death induced by PEITC alone at doses up to 20 μM, but pre‐treatment with DFO blocked the cytotoxic effects of PEITC alone in PANC‐1 cells, demonstrating the iron dependency of the cell death induced by the ITC in pancreatic cancer cells (Kasukabe et al. 2016).

The impairment of redox balance and the alteration of iron metabolism were identified as key characteristics of the anticancer mechanisms of PEITC also in a murine and a human experimental model of osteosarcoma (Lv et al. 2020a, 2020b) (Table 1). Focusing the attention on the ferroptotic mechanism, the treatment with the ITC impaired iron metabolism in a murine and in four human osteosarcoma cell lines starting from 15 μM (Table 1), increasing the level of labile iron (Lv et al. 2020a, 2020b). Labile iron denotes the intracellular nonprotein‐bound iron, meaning redox active iron, which can generate oxygen radicals via the Fenton reactions, thus resulting in oxidative cell damage (Zhang, Xin, et al. 2022). The expression of the iron‐uptake protein TFR1 was upregulated and the expression of iron‐efflux protein FPN, the iron transporter DMT1, the iron storage protein FTH1, and the iron regulatory protein IRP2 were downregulated in human osteosarcoma cells (Lv et al. 2020a). Besides, up‐regulation of mRNA transcript levels of the gene codifying for FTH1 and the iron transporter solute carrier family 40 member 1 (SLC40A1) were recorded while the mRNA of IRP2, the expression of gene codifying for transferrin receptor TFRC and for SLC11A2 all decreased (Lv et al. 2020a). A similar regulation at transcription level of iron regulating genes was observed in K7M2 murine cells (Lv et al. 2020b) (Table 1). The abovementioned described expression of protein and mRNA contributes to the increase in both total and liable iron, thus in the induction of ferroptosis in in vitro models. Moreover, cell treatment with PEITC for 24 h increased lipid peroxidation in human osteosarcoma cells, confirmed by the increase in MDA, one of the main products of lipid peroxidation. PEITC also decreased the ratio between GSH and oxidized GSH (GSH/GSSG) and lowered the expression of GPX4, both leading to ROS accumulation, lipid peroxidation, and oxidative stress in human osteosarcoma cells (Lv et al. 2020a). These findings were confirmed in vivo (Table 1). In particular, 4 weeks old male BALB/c nude mice xenografted with MNNG/HOS human osteosarcoma cells were intragastrically administered with 30 mg/kg/die PEITC in 10% sesame‐seed oil with saline for 24 days. Tumor lysates were then analyzed. PEITC treated group showed decreased FPN and DMT1 expression and increased TFR1 expression compared to the control group. Furthermore, the expression of GPX4 was downregulated, consistently with the results obtained in vitro (Lv et al. 2020a). Tumor dimension was reduced in PEITC‐treated mice. In a syngeneic orthotopic osteosarcoma mouse model, PEITC was administered at three different doses: 30, 60 or 90 mg/kg/die for 24 consecutive days. Interestingly, the reduction of tumor volume was recorded in the groups administered with 30 and 60 mg/kg PEITC compared to the control group; higher doses abolished its anticancer effect (Lv et al. 2020b). Moreover, the pro‐oxidant effect observed at higher doses may affect not only cancer cells but also normal cells, compromising the selectivity of the ITC (Hać et al. 2020; Trachootham et al. 2006).

### 4.2 Sulforaphane

Ferroptosis in osteosarcoma models was also demonstrated for sulforaphane (Figure 2, **2**) (Zou et al. 2025), as demonstrated using different inhibitors of cell death (Table 1). Ferrostatin‐1, DFO, and liproxstatin‐1 substantially rescued the cells from sulforaphane‐induced cell death, indicating that ferroptosis was the predominant cell death induced by sulforaphane. Moreover, sulforaphane treatment elevated ROS levels, decreased GPX4 expression, induced lipid peroxidation a, and depleted GSH, which was dependent on decreased levels of SLC7A11. Morphological changes of osteosarcoma cells observed after sulforaphane treatment were consistent with ferroptotic cell death and included altered mitochondrial structure, increased mitochondrial membrane density, and reduced or absent mitochondrial cristae. Mechanically, sulforaphane directly targeted p62, the multifunctional adaptor protein involved in autophagy (Kumar et al. 2022). In particular, it enhanced p62/SLC7A11protein‐protein interaction, thereby promoting the lysosomal degradation of SLC7A11 and triggering ferroptosis (Zou et al. 2025). The anticancer effects of sulforaphane mediated by ferroptosis were confirmed in a subcutaneous and an intratibial osteosarcoma xenograft model. In particular, BALB/c nude mice subcutaneously injected with 143B cells were treated 6 days post‐injection with 25 or 50 mg/kg sulforaphane every 2 days for 18 days and then mice were euthanized. Compared to the control group, sulforaphane significantly reduced tumor dimensions. The intratibial xenograft nude mice were randomly assigned to different treatment groups: control, ferrostatin‐1 (2 mg/kg), sulforaphane (50 mg/kg), or sulforaphane + ferrostatin‐1 every 2 days for 16 days and then euthanized. Consistent with the subcutaneous tumor, tumor dimensions reduction was observed in sulforaphane‐treated group. Ferrostatin‐1 partially rescued the anticancer effect of the ITC, confirming the involvement of ferroptosis. Interestingly, in the intratibial xenograft model, sulforaphane also reduced the number of lung metastatic foci, an effect partially reversed by ferrostatin‐1 (Zou et al. 2025). In both models of nude mice, the ferroptosis‐associated anticancer activity of sulforaphane required SLC7A11 downregulation. Of note, in both in vivo models neither difference in body weight between the control and sulforaphane‐treated groups, nor pathological issues in the major organs examined (liver, kidney, heart, spleen, and lung) were observed, showing no evidence of toxicity associated with the treatment (Zou et al. 2025).

Sulforaphane, well known for its remarkable apoptotic activity in leukemia cells (Fimognari et al. 2014), was found to trigger ferroptosis in acute myeloid leukemia cell lines (Greco et al. 2021) (Table 1). Interestingly, the treatment concentration of the ITC influenced the cell death mechanisms triggered by sulforaphane, which at low concentrations evoked apoptosis and at the highest concentrations ferroptosis. In particular, pre‐treatment with the pan‐caspases inhibitor Z‐VAD totally rescued cell viability at 25 μM but did not affect cell viability at 50 μM, suggesting the involvement of another mechanism of cell death. Besides, at the highest tested concentration (50 μM), pre‐treatment with ferrostatin‐1 induced a partial recovery of cell viability and a switch in the type of cell death from necrosis to apoptosis, confirming the contribution of ferroptosis in leukemia cell deaths. At a concentration of 50 μM, ferroptosis was induced through depletion of GSH, down regulation of GPX4, and induction of lipid peroxidation (Greco et al. 2021).

Sirtuin‐3 (SIRT3) modulates mitochondrial ROS generation and activates AMPK (AMP‐activated protein kinase), which in turn inhibits mTOR signaling. This metabolic shift enhances oxidative stress, promotes lipid peroxidation, and ultimately facilitates iron‐dependent ferroptotic cell death (Han et al. 2020). Through an overexpression of SIRT3, sulforaphane suppressed SLC7A11 expression by activating the AMPK/mTOR pathway in colon cancer cells (Table 1) (Hu et al. 2024). In colon cancer cells, sulforaphane up‐regulated SIRT3, phosphorylated AMPK and mTOR expression, and down‐regulated SLC7A11 in a concentration‐dependent manner starting from 4 μM, leading to ferroptotic cell death. The contribution of the SIRT3/AMPK/mTOR axis in the ferroptosis induced by the ITC was confirmed using inhibitors of AMPK or mTOR (dorsomorphin and rapamycin, respectively) and SIRT3 knockdown cells, which partially reversed the ability of sulforaphane to induce ferroptosis in HCT‐116 cells (Hu et al. 2024). These results confirmed that SIRT3 at least partially contributes to sulforaphane‐induced ferroptosis in colon cancer cells.

Sulforaphane induced ferroptotic cell death also in small‐cell lung cancer cells (Iida et al. 2021). Pre‐treatment with ferrostatin‐1 and DFO recovered cell viability, confirming the ferroptotic effect of the ITC. Moreover, after treatment with sulforaphane, iron levels increased, leading to the accumulation of lipid ROS. Besides, protein and mRNA expression of SLC7A11 was significantly decreased by sulforaphane (20 μM) after 96 h treatment (Table 1), leading to a decrease in intracellular cystine and GSH and, thus, fueling iron‐dependent lipid peroxidation. Interestingly, ferrostatin‐1 also inhibited cell death in cross‐resistant to anthracycline analogs lung cancer cell line (H69AR) (Iida et al. 2021). Thus, sulforaphane may represent an interesting adjuvant anticancer strategy even in tumors that have shown resistance to cytotoxic anticancer drugs.

### 4.3 Synthetic ITCs

**FIGURE 3:**
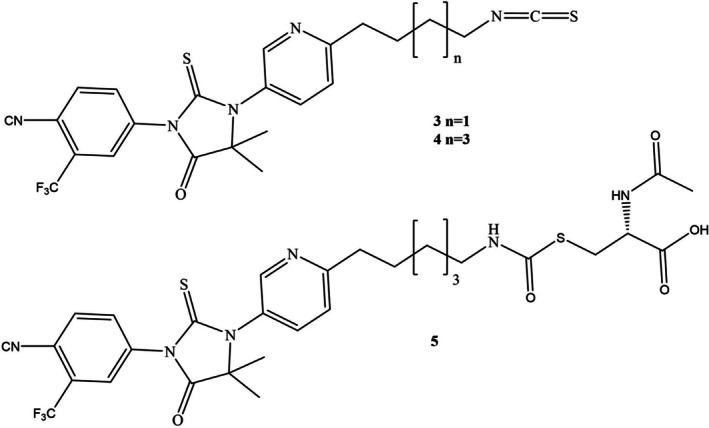
Chemical structure of the ITC‐Ari (**3, 4, 5**).

**FIGURE 4:**
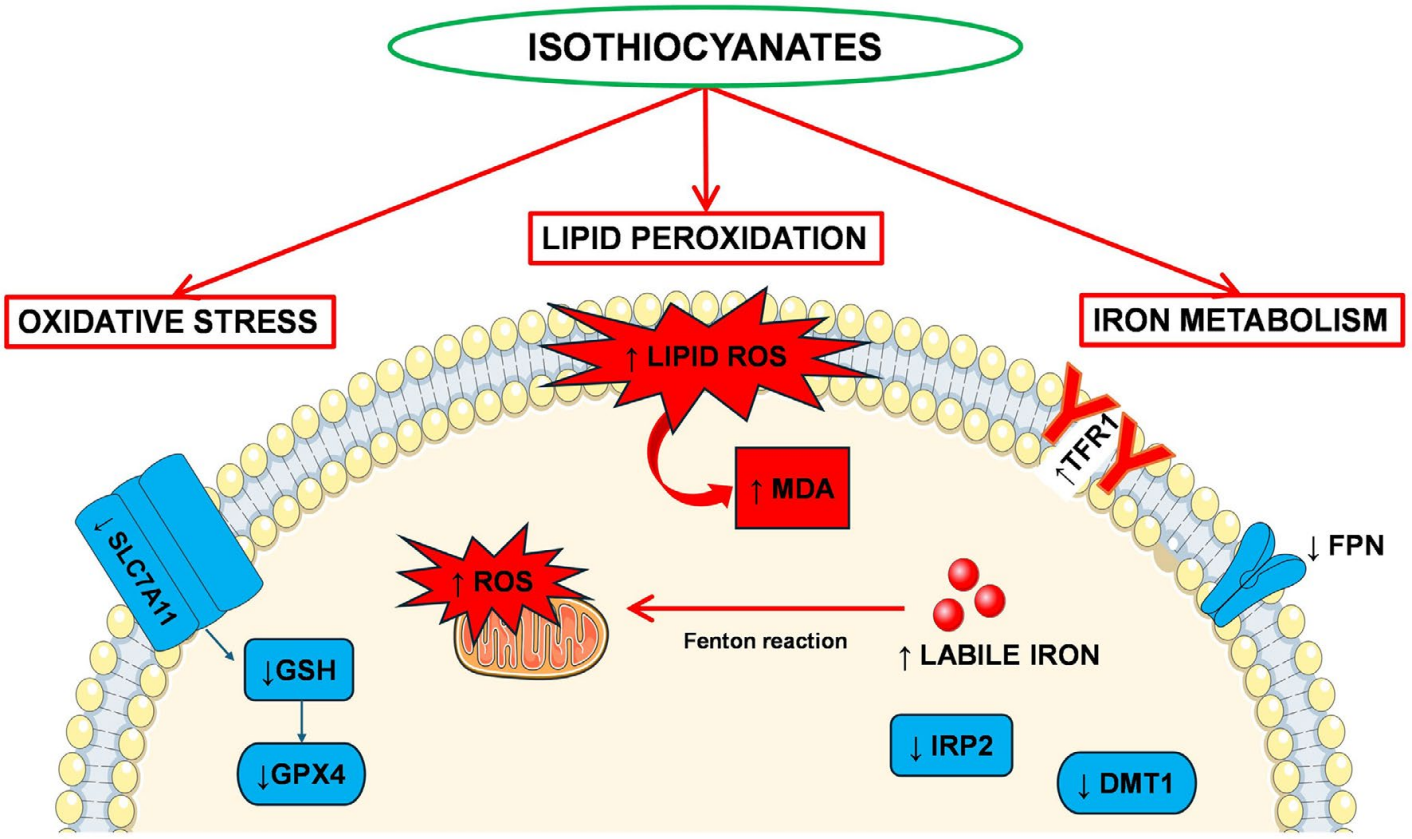
Key ferroptototic mechanisms targeted by ITCs. ITCs promote oxidative stress (depletion of GSH, downregulation of GPX4, inhibition of SLC7A11), disrupting iron metabolism (increased labile iron and TFR1 activity, decreased FPN activity), and enhancing lipid peroxidation (accumulation of lipid ROS and MDA), ultimately causing membrane damage. Red elements indicate induction or upregulation (↑), whereas blue elements indicate inhibition or downregulation (↓). DMT1: Divalent metal transporter; FPN: Ferroportin; IRP2: Iron regulatory protein 2; GPX4: Glutathione peroxidase 4; GSH: Glutathione; MDA: Malondialdehyde; SLC7A11: Solute Carrier Family 7 Member 11; TFR1: Iron‐uptake protein transferrin receptor‐1.

**FIGURE 5:**
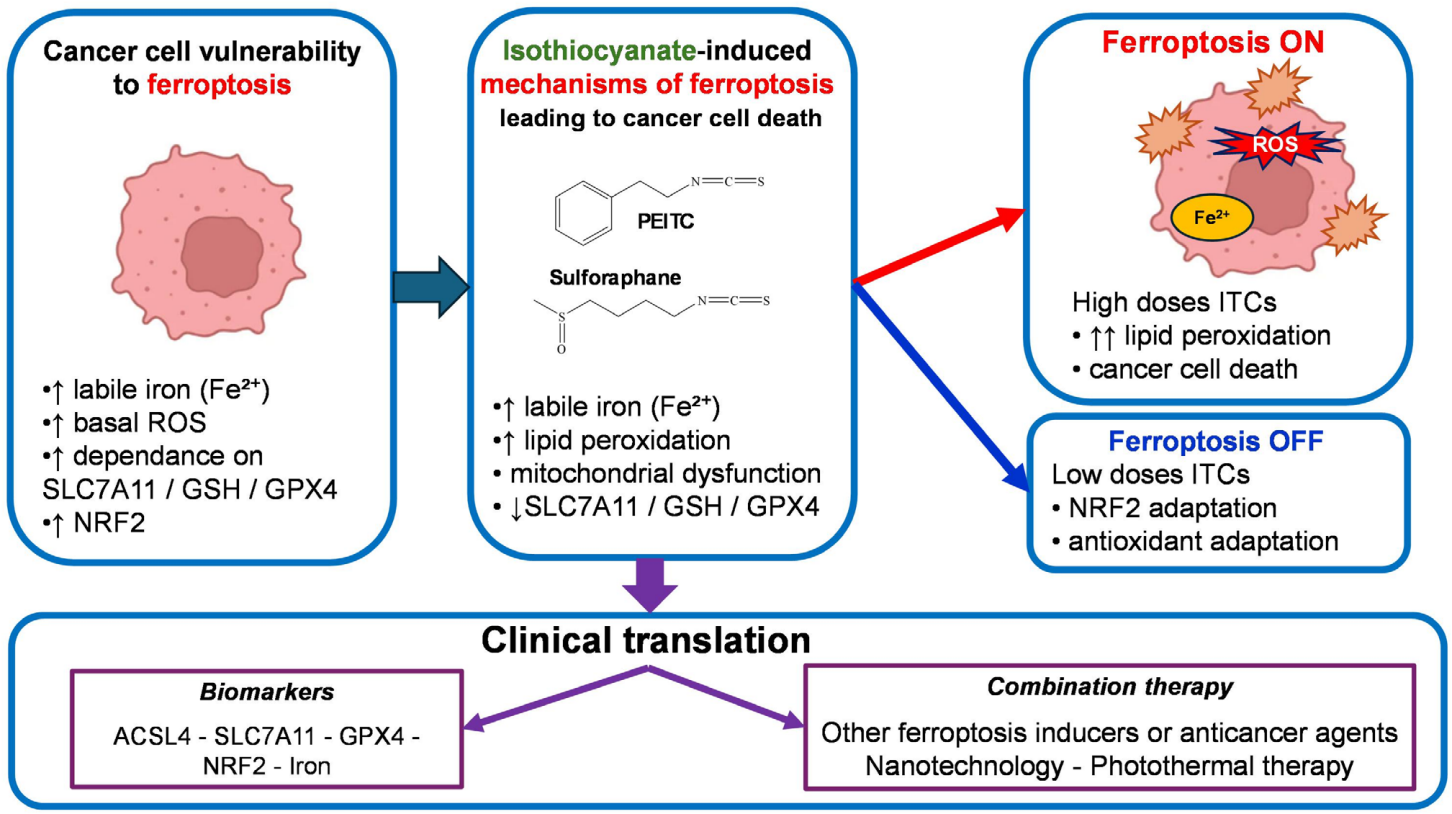
ITCs as ferroptosis inducers in cancer.

A paper showed the ferroptotic anticancer activity of molecules designed and synthesized combining an ITC with a hybrid antagonist of the androgen receptor (Ari) (Figure 3, **3**, **4**, **5**). The ITC‐Ari **5** is an AR‐ligand containing an N‐acetyl cysteine masked AR moiety that releases the parental unconjugated ITC **4** in aqueous solution (Qin et al. 2021).

VCaP cells are a castration‐resistant prostate cancer model characterized by AR overexpression and apoptosis evasion. The ITC‐ARi exhibited cytotoxic effects on VCaP cells (Qin et al. 2021). Despite the higher potency of compound **4**, compound **5** was prioritized for further investigation because its prodrug‐like behavior allowed a more controlled release of the active ITC, ideally resulting in improved cancer selectivity. Indeed, when the cytotoxic activity of the two compounds was compared in LNCaP prostate cancer cells and the non‐cancerous prostatic epithelial RWPE‐1 cells, only compound **5** showed partial selectivity (about 20% difference at the tested concentrations 2.5, 5, and 10 μM), likely due to the gradual release of unconjugated compound **4** that limits abrupt electrophilic insult. The ITC‐ARi **5** induced lipid peroxidation and downregulation of AR. Further depletion of GSH induced by the GSH inhibitor buthionine sulfoximine (BSO) potentiated the anticancer effect of **5**, synergistically increasing its lipid oxidative stress (Table 1). The synergistic effect was mainly due to insufficient lipid damage repair and the accumulation of lipid hydroperoxide. The synergism of ITC‐ARi **5** and BSO was not recorded in non‐transformed prostate RWPE‐1 cells, suggesting a peculiar activity of the combination towards cancer cells. Cells treatment with the iron chelator DFO, vitamin E, or ferrostatin‐1 contrasted the cytotoxic activity of ITC‐ARi/BSO, supporting the induction of ferroptotic cell death by this treatment. Moreover, treatment with the specific inhibitor of Hmox1 Zinc(II) protoporphyrin IX inhibited cell death, highlighting the involvement of the iron‐dependent cell death in the recorded cytotoxic effects (Qin et al. 2021). The ability of ITC‐ARi **5** to trigger a different mechanism of cell death, such as ferroptosis, and to downregulate AR makes this molecule a good lead compound to fight castration‐resistant prostate cancer.

## 5 Discussion

The experimental evidence summarized in the main body of this review indicates that ITCs can trigger ferroptosis through multiple, partly convergent mechanisms (Figure 4).

PEITC primarily promotes ferroptosis by disrupting iron homeostasis and enhancing lipid ROS generation, increasing the LIP, and amplifying oxidative damage at the membrane level (Lv et al. 2020a, 2020b). Sulforaphane mainly targets the Xc^−^–GSH–GPX4 axis, downregulating SLC7A11, depleting GSH, and suppressing GPX4 expression, thereby lowering the antioxidant threshold that prevents ferroptotic execution (Greco et al. 2021; Hu et al. 2024; Iida et al. 2021; Zou et al. 2025). ITCs, as redox active compounds, elicit a biphasic dose–response: at low concentrations, adaptive cytoprotective mechanisms are activated, while at higher doses, ITCs overwhelm cellular antioxidant capacity and promote oxidative cell death (Wang, Li, et al. 2024). In this context, ferroptosis emerges as a critical downstream outcome when lipid peroxidation and iron‐dependent oxidative stress exceed cellular buffering systems. The hormetic behavior of ITCs has important implications for ferroptosis‐based cancer therapy (Wang, Li, et al. 2024). At low doses, ITCs primarily induce antioxidant and metabolic adaptation, limiting lipid peroxidation and conferring resistance to ferroptosis: they induce the activation of the Nrf2 signaling pathway, thereby strengthening endogenous antioxidant systems and increasing resistance to oxidative damage. This is probably due to the ability of ITCs to release hydrogen sulfide (H_2_S) through a nucleophilic reaction with L‐cysteine (Lin et al. 2019). Indeed, ITCs and H₂S share an overlap in their mechanisms of action, particularly in redox regulation and ferroptosis control (Citi et al. 2022). Both promote antioxidant defenses, enhance GSH homeostasis, and modulate lipid peroxidation processes (Citi et al. 2022). Furthermore, H_2_S is an endogenous gasotransmitter that plays a central role in redox homeostasis and ferroptosis regulation. Endogenously produced by enzymes such as cystathionine gamma‐lyase and cystathionine beta‐synthase (Cirino et al. 2023), H₂S has emerged as a key inhibitor of ferroptosis by stabilizing system Xc^−^, enhancing GPX4 expression, and suppressing key ferroptosis‐related pathways such as ALOX12 acetylation (Chen, Bu, et al. 2021; Wang et al. 2021). In contrast, higher or sustained exposures to ITCs can disrupt redox homeostasis, impair GSH‐ and GPX4‐dependent defenses, and favor ferroptotic cell death (Greco et al. 2021). Most of the studies presented here focus on relatively high concentrations of ITCs, often exceeding 10–20 μM in vitro, where ferroptotic cell death is readily observed. As a result, the transition between cytoprotective and cytotoxic responses is usually inferred rather than experimentally demonstrated. Besides, most studies lack a systematic assessment of ITCs selectivity against cancer cells, making it difficult to distinguish between cancer‐selective ferroptosis and nonspecific cytotoxic effects on normal cells. Moreover, although NRF2 signaling is widely acknowledged as a central regulator of redox homeostasis and ferroptosis resistance (Das et al. 2013; Jaganjac et al. 2020), the reviewed articles do not evaluate NRF2 nuclear translocation, transcriptional activity, or the expression of NRF2 target genes in parallel with ferroptotic markers, nor do they employ genetic or pharmacological NRF2 modulation to establish causality. This represents a significant gap, particularly given that ITCs are well‐known NRF2 activators at low doses and that NRF2‐driven upregulation of SLC7A11, GPX4, and FSP1 could antagonize ferroptotic execution (Jaganjac et al. 2020). Consequently, whether ferroptosis induction by ITCs reflects true circumvention of NRF2‐mediated defenses or simply occurs under conditions where these defenses are overwhelmed remains unresolved. Addressing these gaps will be essential to link the strong ferroptotic activity observed in experimental models with the pronounced hormetic behavior of ITCs and to define rational dosing strategies capable of minimizing adaptive resistance while maximizing therapeutic efficacy.

Osteosarcoma has emerged as a relevant experimental model to investigate ferroptosis induced by ITCs, owing to its intrinsic sensitivity to iron‐dependent oxidative stress and lipid peroxidation (Borović Šunjić et al. 2024). Ferroptosis contributes together with the other mechanisms of cell death, such as apoptosis and autophagy, to the anticancer effects of PEITC and sulforaphane in osteosarcoma models both in vitro and in vivo (Lv et al. 2020a, 2020b; Zou et al. 2025). Mitochondrial dysfunction is a key driver of ferroptosis. New research has shown that lipid peroxidation specifically inside the mitochondria is a critical early event that triggers ferroptotic cell death, as it promotes iron‐dependent lipid peroxidation and redox imbalance (Lyamzaev et al. 2023). Zhen and colleagues further investigated the role of mitochondria in PEITC‐induced cell death in osteosarcoma cells (Zhen et al. 2023). PEITC promoted cytosolic, lipid, and mitochondrial ROS accumulation, accompanied by marked mitochondrial remodeling and dysfunction. Moreover, PEITC induced dynamic changes in mitochondrial membrane potential, impaired respiratory chain activity, and caused transient ATP elevation followed by depletion, ultimately reducing cell proliferation. Notably, mitochondrial DNA–depleted (ρ^0^) cells displayed reduced sensitivity to PEITC, indicating that mitochondrial function critically contributes to PEITC‐induced oxidative cell death (Zhen et al. 2023). Although ferroptosis was not explicitly investigated, PEITC‐induced mitochondrial dysfunction, lipid and mitochondrial ROS accumulation, and metabolic alterations are highly consistent with key morphological and molecular features associated with ferroptotic cell death.

An emerging and promising aspect is the combination of ITCs with other natural or synthetic compounds or advanced formulations to counteract cancer progression. The combination of PEITC with cotylenin synergistically induced cell death at concentrations where either compound alone was ineffective (Kasukabe et al. 2016). The synthetic derivative ITCs‐ARi **5** showed that simultaneously targeting AR‐driven survival pathways and ferroptosis‐related antioxidant defenses may limit the adaptive capacity of cancer cells and reduce reliance on apoptosis‐dependent cell death (Qin et al. 2021). Together, these findings suggest that combining ITCs with complementary natural or synthetic agents can disrupt survival signaling and redox homeostasis and enhance their anticancer efficacy. However, the lack of in vivo evidence remains a critical gap in validating the translational relevance of these combination strategies.

Chemodynamic therapy is an anticancer strategy that exploits tumor‐specific chemical conditions to catalytically generate cytotoxic ROS through Fenton‐like reactions, leading to oxidative stress–mediated tumor cell death. The therapeutic efficacy of chemodynamic therapy is frequently hampered by insufficient endogenous H_2_O_2_ levels, inefficient catalytic activity, and the strong antioxidant capacity of the tumor microenvironment (Shen et al. 2026). For this reason, chemodynamic therapy is often combined with (i) photothermal therapy to accelerate reaction kinetics that boosts ROS generation; (ii) redox modulators to deplete GSH; (iii) ferroptosis inducers to amplify lipid peroxidation; or (iv) nanomaterials to improve tumor targeting (Zhu et al. 2025). A very recent study underscores the therapeutic potential of integrating photothermal and chemodynamic modalities to overcome tumor antioxidant defenses. In particular, ruthenium‐procyanidin nanoparticles were coencapsulated with PEITC in a sodium alginate hydrogel matrix (Ye et al. 2025). In this multifunctional platform, PEITC could deplete intracellular GSH, thereby weakening redox buffering capacity and sensitizing tumor cells to ROS‐mediated damage. This redox modulation could markedly enhance the chemodynamic efficacy of the ruthenium‐based system, allowing photothermally accelerated Fenton‐like reactions to generate cytotoxic radicals more efficiently within the tumor microenvironment (Ye et al. 2025). The combined chemo‐phototherapeutic approach not only improved local tumor ablation through controlled photothermal heating but also amplified oxidative stress beyond the adaptive threshold of cancer cells, promoting the accumulation of lipid hydroperoxides and engaging ferroptosis alongside mitochondrial dysfunction and apoptotic signaling. Theoretically, such a multifunctional platform may also help overcome adaptive antioxidant responses, including NRF2‐mediated defenses, that limit the efficacy of redox‐active agents when used as monotherapies.

Further evidence supporting the use of PEITC as a potent ferroptosis‐sensitizing agent in combination‐based therapeutic strategies emerged in a study by Wang, Zhong, et al. (2024), where the polyphenol epigallocatechin gallate was combined with PEITC to form the so‐called EFP nanocapsules, which were further integrated into microneedle patches obtaining the so‐called all‐in‐one EFP@MNs (Wang, Zhong, et al. 2024). Lipophagy is a selective autophagic process that degrades lipid droplets to release free fatty acids, thereby providing substrates that fuel lipid peroxidation and promote ferroptosis. Although lipophagy increases the availability of PUFAs required for lipid peroxidation, its therapeutic impact is often limited by metabolic rerouting of released lipids and the activation of antioxidant defense mechanisms (Kounakis et al. 2019). Epigallocatechin gallate is a lipophagy inducer. By acting as a robust inducer of oxidative stress, PEITC may synergize with Fe^2+^‐mediated lipid peroxidation once sufficient free fatty acids are released through lipophagy, thereby overcoming a major metabolic limitation of ferroptosis‐driven tumor eradication (Wang, Zhong, et al. 2024). Wang, Zhong, et al. (2024) demonstrated that EFP nanocapsules caused metabolic alterations and induced lipophagy, thus favoring the production of lipid peroxide and ferroptosis. The combination of the EFP@MNs platform with photothermal therapy further amplified lipid peroxide accumulation and ferroptotic execution.

Despite the extensive preclinical evidence supporting the ferroptosis‐inducing potential of ITCs, clinical translation remains limited. To date, only a small number of early‐phase clinical trials have investigated the efficacy, safety, and tolerability of ITCs, primarily focusing on sulforaphane.

Notably, a phase II study (single arm trial) (NCT01228084) demonstrated that daily treatment with 200 μmoles of sulforaphane‐rich broccoli sprout extracts for a maximum period of 20 weeks does not lead to a large reduction in prostate‐specific antigen levels in most patients with recurrent prostate cancer. Despite the lack of efficacy, treatment with 200 μmoles of sulforaphane was well‐tolerated with no Grade 3 or 4 adverse effects. The most common adverse events were gastrointestinal disorders of Grade 1, indicating that the dose tested is well tolerated. The limited anticancer activity observed may be due, at least in part, to low sulforaphane dosing and suboptimal treatment schedules (Alumkal et al. 2015).

Similarly, a phase I randomized, parallel‐assigned trial assessing the biological effects of a high‐sulforaphane broccoli sprout extract in normal prostate tissue (NCT00946309) showed that the administration of 100 μmoles sulforaphane every other day for 5 weeks in healthy men was associated with gastrointestinal disorders of Grade 1 or 2, with no serious adverse events reported.

Recently, the safety and tolerability of an enteric‐coated tablet formulation of synthetic form of *d,l* sulforaphane (SFX‐01) were evaluated in a randomized, double‐blind, placebo‐controlled, dose‐escalation study [300 mg once daily (46.2 mg sulforaphane, 260.59 μmoles); 300 mg twice daily or 600 mg once daily (92.4 mg sulforaphane, 521.18 μmoles)] over 7 days in healthy male. Adverse events occurred in 94% of participants who received SFX‐01 and were most commonly gastrointestinal events, which were mild in severity and related to treatment (Clack et al. 2025).

In summary, the clinical trials were not designed to investigate ferroptosis‐related endpoints, nor did they assess redox biomarkers, lipid peroxidation, NRF2 activation status, or iron metabolism. Consequently, the role of ferroptosis triggered by sulforaphane in humans remains unknown. However, the available data showed a safe profile of sulforaphane. The integration of mechanistic biomarkers into future clinical trial designs will be essential to bridge the gap between preclinical ferroptosis‐based evidence and clinical application of ITCs.

## 6 Conclusion and Future Directions

In conclusion, ITCs emerge as versatile inducers of ferroptosis, acting through distinct and compound‐specific mechanisms. While PEITC predominantly promotes ferroptotic cell death by enhancing lipid ROS generation and disrupting iron homeostasis, sulforaphane mainly sensitizes cells to ferroptosis through inhibition of the system Xc^−^ antiporter (Figure 5).

ITCs are generally characterized by a favorable safety profile in vivo and in humans and a partial selectivity toward cancer cell lines, which enhance their attractiveness as therapeutic agents. However, the pronounced hormetic behavior of ITCs, whereby concentration and exposure time dictate cytoprotective versus cytotoxic outcomes, highlights the necessity for precise therapeutic optimization. Future research should focus on defining dose‐ and tumor‐specific‐dependent determinants that govern the engagement of ferroptosis relative to other cell death pathways across different tumor types. In this regard, the evaluation of predictive biomarkers of ferroptotic sensitivity, such as ACSL4 expression, SLC7A11/GPX4 status, iron‐handling proteins, and NRF2 activity, will be critical to predict tumor cell susceptibility to ferroptosis for future clinical application and optimization of personalized therapy. Moreover, the combination of ITCs with ferroptosis inducers, conventional chemotherapeutics, targeted agents, or photothermal therapy represents a promising strategy to overcome resistance while limiting toxicity to normal tissues. Advances in nanotechnology may further enhance ITCs bioavailability, stability, and tumor selectivity, facilitating clinical translation. A deeper understanding of the multilayered regulatory networks governing ITC‐induced ferroptosis in preclinical studies, coupled with clinical trials, will be essential to harness the full therapeutic potential of ITCs as rational ferroptosis‐modulating agents in oncology.

## Author Contributions

**Valentina Citi:** conceptualization, funding acquisition, writing – original draft, writing – review and editing. **Renda Chahna:** writing – original draft. **Francesca Maffei:** investigation, writing – original draft, writing – review and editing, data curation. **Alma Martelli:** writing – review and editing. **Vincenzo Calderone:** writing – review and editing. **Carmela Fimognari:** writing – review and editing. **Eleonora Turrini:** conceptualization, investigation, funding acquisition, writing – original draft, writing – review and editing, data curation.

## Funding

This work was supported by Ministero dell'Università e della Ricerca PRIN 2022 grant (project code: 2022N3FLP3).

## Conflicts of Interest

The authors declare no conflicts of interest.

## Data Availability

Data sharing not applicable to this article as no datasets were generated or analysed during the current study.

## References

[ptr70389-bib-0001] Alumkal, J. J. , R. Slottke , J. Schwartzman , et al. 2015. “A Phase II Study of Sulforaphane‐Rich Broccoli Sprout Extracts in Men With Recurrent Prostate Cancer.” Investigational New Drugs 33: 480–489. 10.1007/s10637-014-0189-z. 25431127 PMC4390425

[ptr70389-bib-0002] Asma, S. T. , U. Acaroz , K. Imre , et al. 2022. “Natural Products/Bioactive Compounds as a Source of Anticancer Drugs.” Cancers (Basel) 14: 6203. 10.3390/cancers14246203. 36551687 PMC9777303

[ptr70389-bib-0003] Bersuker, K. , J. M. Hendricks , Z. Li , et al. 2019. “The CoQ Oxidoreductase FSP1 Acts Parallel to GPX4 to Inhibit Ferroptosis.” Nature 575: 688–692. 10.1038/s41586-019-1705-2. 31634900 PMC6883167

[ptr70389-bib-0004] Borović Šunjić, S. , M. Jaganjac , J. Vlainić , M. Halasz , and N. Žarković . 2024. “Lipid Peroxidation‐Related Redox Signaling in Osteosarcoma.” International Journal of Molecular Sciences 25: 4559. 10.3390/ijms25084559. 38674143 PMC11050283

[ptr70389-bib-0005] Bray, F. , M. Laversanne , H. Sung , et al. 2024. “Global Cancer Statistics 2022: GLOBOCAN Estimates of Incidence and Mortality Worldwide for 36 Cancers in 185 Countries.” CA: a Cancer Journal for Clinicians 74: 229–263. 10.3322/caac.21834. 38572751

[ptr70389-bib-0006] Chen, S. , D. Bu , J. Zhu , et al. 2021. “Endogenous Hydrogen Sulfide Regulates xCT Stability Through Persulfidation of OTUB1 at Cysteine 91 in Colon Cancer Cells.” Neoplasia 23: 461–472. 10.1016/j.neo.2021.03.009. 33878705 PMC8081877

[ptr70389-bib-0007] Chen, X. , R. Kang , G. Kroemer , and D. Tang . 2021. “Broadening Horizons: The Role of Ferroptosis in Cancer.” Nature Reviews. Clinical Oncology 18: 280–296. 10.1038/s41571-020-00462-0. 33514910

[ptr70389-bib-0008] Chen, Z. , W. Wang , S. R. Abdul Razak , T. Han , N. H. Ahmad , and X. Li . 2023. “Ferroptosis as a Potential Target for Cancer Therapy.” Cell Death & Disease 14: 1–15. 10.1038/s41419-023-05930-w. 37488128 PMC10366218

[ptr70389-bib-0009] Choudhari, A. S. , P. C. Mandave , M. Deshpande , P. Ranjekar , and O. Prakash . 2020. “Phytochemicals in Cancer Treatment: From Preclinical Studies to Clinical Practice.” Frontiers in Pharmacology 10: 1614. 10.3389/fphar.2019.01614. 32116665 PMC7025531

[ptr70389-bib-0010] Cirino, G. , C. Szabo , and A. Papapetropoulos . 2023. “Physiological Roles of Hydrogen Sulfide in Mammalian Cells, Tissues, and Organs.” Physiological Reviews 103: 31–276. 10.1152/physrev.00028.2021. 35435014

[ptr70389-bib-0011] Citi, V. , E. Piragine , V. Calderone , and A. Martelli . 2022. “Hydrogen Sulfide: The Hidden Player of Isothiocyanates Pharmacology.” In Hydrogen Sulfide, 261–292. John Wiley & Sons, Ltd. 10.1002/9781119799900.ch11.

[ptr70389-bib-0012] Clack, G. , C. Moore , L. Ruston , et al. 2025. “A Phase 1 Randomized, Placebo‐Controlled Study Evaluating the Safety, Tolerability, and Pharmacokinetics of Enteric‐Coated Stabilized Sulforaphane (SFX‐01) in Male Participants.” Advances in Therapy 42: 216–232. 10.1007/s12325-024-03018-1. 39520658 PMC11782309

[ptr70389-bib-0013] Dai, E. , L. Han , J. Liu , et al. 2020. “Ferroptotic Damage Promotes Pancreatic Tumorigenesis Through a TMEM173/STING‐Dependent DNA Sensor Pathway.” Nature Communications 11: 6339. 10.1038/s41467-020-20154-8. PMC7732843 33311482

[ptr70389-bib-0014] Das, B. N. , Y.‐W. Kim , and Y.‐S. Keum . 2013. “Mechanisms of Nrf2/Keap1‐Dependent Phase II Cytoprotective and Detoxifying Gene Expression and Potential Cellular Targets of Chemopreventive Isothiocyanates.” Oxidative Medicine and Cellular Longevity 2013: 839409. 10.1155/2013/839409. 23781297 PMC3679808

[ptr70389-bib-0015] Dixon, S. J. , K. M. Lemberg , M. R. Lamprecht , et al. 2012. “Ferroptosis: An Iron‐Dependent Form of Nonapoptotic Cell Death.” Cell 149: 1060–1072. 10.1016/j.cell.2012.03.042. 22632970 PMC3367386

[ptr70389-bib-0016] Dixon, S. J. , D. N. Patel , M. Welsch , et al. 2014. “Pharmacological Inhibition of Cystine‐Glutamate Exchange Induces Endoplasmic Reticulum Stress and Ferroptosis.” eLife 3: e02523. 10.7554/eLife.02523. 24844246 PMC4054777

[ptr70389-bib-0017] Fimognari, C. , E. Turrini , P. Sestili , et al. 2014. “Antileukemic Activity of Sulforaphane in Primary Blasts From Patients Affected by Myelo‐ and Lympho‐Proliferative Disorders and in Hypoxic Conditions.” PLoS One 9: e101991. 10.1371/journal.pone.0101991. 25019218 PMC4096754

[ptr70389-bib-0018] Greco, G. , M. Schnekenburger , E. Catanzaro , et al. 2021. “Discovery of Sulforaphane as an Inducer of Ferroptosis in U‐937 Leukemia Cells: Expanding Its Anticancer Potential.” Cancers (Basel) 14: 76. 10.3390/cancers14010076. 35008240 PMC8750507

[ptr70389-bib-0019] Grube, J. , M. M. Woitok , A. Mohs , et al. 2022. “ACSL4‐Dependent Ferroptosis Does Not Represent a Tumor‐Suppressive Mechanism but ACSL4 Rather Promotes Liver Cancer Progression.” Cell Death & Disease 13: 704. 10.1038/s41419-022-05137-5. 35963845 PMC9376109

[ptr70389-bib-0021] Hać, A. , J. Brokowska , E. Rintz , M. Bartkowski , G. Węgrzyn , and A. Herman‐Antosiewicz . 2020. “Mechanism of Selective Anticancer Activity of Isothiocyanates Relies on Differences in DNA Damage Repair Between Cancer and Healthy Cells.” European Journal of Nutrition 59: 1421–1432. 10.1007/s00394-019-01995-6. 31123866 PMC7230056

[ptr70389-bib-0022] Han, D. , L. Jiang , X. Gu , et al. 2020. “SIRT3 Deficiency Is Resistant to Autophagy‐Dependent Ferroptosis by Inhibiting the AMPK/mTOR Pathway and Promoting GPX4 Levels.” Journal of Cellular Physiology 235: 8839–8851. 10.1002/jcp.29727. 32329068

[ptr70389-bib-0023] Hashem, S. , T. A. Ali , S. Akhtar , et al. 2022. “Targeting Cancer Signaling Pathways by Natural Products: Exploring Promising Anti‐Cancer Agents.” Biomedicine & Pharmacotherapy 150: 113054. 10.1016/j.biopha.2022.113054. 35658225

[ptr70389-bib-0024] Hu, B. , P. Cao , J.‐H. Wang , W. Feng , Y. Zhang , and H. Yang . 2024. “Sulforaphane Triggers Sirtuin 3‐Mediated Ferroptosis in Colorectal Cancer Cells via Activating the Adenosine 5′‐Monophosphate (AMP)‐Activated Protein Kinase/ Mechanistic Target of Rapamycin Signaling Pathway.” Human & Experimental Toxicology 43: 9603271241266106. 10.1177/09603271241266106. 39291655

[ptr70389-bib-0025] Iida, Y. , M. Okamoto‐Katsuyama , S. Maruoka , et al. 2021. “Effective Ferroptotic Small‐Cell Lung Cancer Cell Death From SLC7A11 Inhibition by Sulforaphane.” Oncology Letters 21: 71. 10.3892/ol.2020.12332. 33365082 PMC7716721

[ptr70389-bib-0026] Jaganjac, M. , L. Milkovic , S. B. Sunjic , and N. Zarkovic . 2020. “The NRF2, Thioredoxin, and Glutathione System in Tumorigenesis and Anticancer Therapies.” Antioxidants (Basel) 9: 1151. 10.3390/antiox9111151. 33228209 PMC7699519

[ptr70389-bib-0027] Jiang, L. , N. Kon , T. Li , et al. 2015. “Ferroptosis as a p53‐Mediated Activity During Tumour Suppression.” Nature 520: 57–62. 10.1038/nature14344. 25799988 PMC4455927

[ptr70389-bib-0028] Jiang, X. , B. R. Stockwell , and M. Conrad . 2021. “Ferroptosis: Mechanisms, Biology and Role in Disease.” Nature Reviews. Molecular Cell Biology 22: 266–282. 10.1038/s41580-020-00324-8. 33495651 PMC8142022

[ptr70389-bib-0029] Joković, N. , S. Pešić , J. Vitorović , A. Bogdanović , J. Sharifi‐Rad , and D. Calina . 2025. “Glucosinolates and Their Hydrolytic Derivatives: Promising Phytochemicals With Anticancer Potential.” Phytotherapy Research 39: 1035–1089. 10.1002/ptr.8419. 39726346

[ptr70389-bib-0030] Kagan, V. E. , G. Mao , F. Qu , et al. 2017. “Oxidized Arachidonic and Adrenic PEs Navigate Cells to Ferroptosis.” Nature Chemical Biology 13: 81–90. 10.1038/nchembio.2238. 27842066 PMC5506843

[ptr70389-bib-0032] Kasukabe, T. , Y. Honma , J. Okabe‐Kado , Y. Higuchi , N. Kato , and S. Kumakura . 2016. “Combined Treatment With Cotylenin A and Phenethyl Isothiocyanate Induces Strong Antitumor Activity Mainly Through the Induction of Ferroptotic Cell Death in Human Pancreatic Cancer Cells.” Oncology Reports 36: 968–976. 10.3892/or.2016.4867. 27375275

[ptr70389-bib-0033] Kounakis, K. , M. Chaniotakis , M. Markaki , and N. Tavernarakis . 2019. “Emerging Roles of Lipophagy in Health and Disease.” Frontiers in Cell and Developmental Biology 7: 185. 10.3389/fcell.2019.00185. 31552248 PMC6746960

[ptr70389-bib-0034] Kumar, A. V. , J. Mills , and L. R. Lapierre . 2022. “Selective Autophagy Receptor p62/SQSTM1, a Pivotal Player in Stress and Aging.” Frontiers in Cell and Development Biology 10: 793328. 10.3389/fcell.2022.793328. PMC8883344 35237597

[ptr70389-bib-0035] Labudda, M. , A. Rybarczyk‐Płońska , K. A. Sobieszek , et al. 2026. “The Chemopreventive and Anticancer Potential of Glucosinolates and Their Hydrolysis Products From Cruciferous Vegetables.” Nutrients 18: 751. 10.3390/nu18050751. 41829922 PMC12987301

[ptr70389-bib-0036] Lei, G. , L. Zhuang , and B. Gan . 2022. “Targeting Ferroptosis as a Vulnerability in Cancer.” Nature Reviews. Cancer 22: 381–396. 10.1038/s41568-022-00459-0. 35338310 PMC10243716

[ptr70389-bib-0037] Leng, G. , B. Duan , J. Liu , et al. 2024. “The Advancements and Prospective Developments in Anti‐Tumor Targeted Therapy.” Neoplasia 56: 101024. 10.1016/j.neo.2024.101024. 39047659 PMC11318541

[ptr70389-bib-0038] Li, J. , F. Cao , H.‐L. Yin , et al. 2020. “Ferroptosis: Past, Present and Future.” Cell Death & Disease 11: 88. 10.1038/s41419-020-2298-2. 32015325 PMC6997353

[ptr70389-bib-0039] Li, L. , T. Shan , D. Zhang , and F. Ma . 2024. “Nowcasting and Forecasting Global Aging and Cancer Burden: Analysis of Data From the GLOBOCAN and Global Burden of Disease Study.” Journal of the National Cancer Center 4: 223–232. 10.1016/j.jncc.2024.05.002. 39281725 PMC11401500

[ptr70389-bib-0040] Lin, Y. , X. Yang , Y. Lu , D. Liang , and D. Huang . 2019. “Isothiocyanates as H2S Donors Triggered by Cysteine: Reaction Mechanism and Structure and Activity Relationship.” Organic Letters 21: 5977–5980. 10.1021/acs.orglett.9b02117. 31318571

[ptr70389-bib-0041] Liu, M. , X.‐Y. Kong , Y. Yao , et al. 2022. “The Critical Role and Molecular Mechanisms of Ferroptosis in Antioxidant Systems: A Narrative Review.” Annals of Translational Medicine 10: 368. 10.21037/atm-21-6942. 35434035 PMC9011221

[ptr70389-bib-0042] Lv, H. , C. Zhen , J. Liu , and P. Shang . 2020a. “β‐Phenethyl Isothiocyanate Induces Cell Death in Human Osteosarcoma Through Altering Iron Metabolism, Disturbing the Redox Balance, and Activating the MAPK Signaling Pathway.” Oxidative Medicine and Cellular Longevity 2020: 5021983. 10.1155/2020/5021983. 32322335 PMC7160723

[ptr70389-bib-0043] Lv, H.‐H. , C.‐X. Zhen , J.‐Y. Liu , and P. Shang . 2020b. “PEITC Triggers Multiple Forms of Cell Death by GSH‐Iron‐ROS Regulation in K7M2 Murine Osteosarcoma Cells.” Acta Pharmacologica Sinica 41: 1119–1132. 10.1038/s41401-020-0376-8. 32132657 PMC7468252

[ptr70389-bib-0044] Lyamzaev, K. G. , A. A. Panteleeva , R. A. Simonyan , A. V. Avetisyan , and B. V. Chernyak . 2023. “The Critical Role of Mitochondrial Lipid Peroxidation in Ferroptosis: Insights From Recent Studies.” Biophysical Reviews 15: 875–885. 10.1007/s12551-023-01126-w. 37974984 PMC10643799

[ptr70389-bib-0045] Na, X. , L. Li , D. Liu , J. He , L. Zhang , and Y. Zhou . 2024. “Natural Products Targeting Ferroptosis Pathways in Cancer Therapy (Review).” Oncology Reports 52: 1–21. 10.3892/or.2024.8782. PMC11292301 39054952

[ptr70389-bib-0046] Pedrera, L. , R. A. Espiritu , U. Ros , et al. 2021. “Ferroptotic Pores Induce Ca2+ Fluxes and ESCRT‐III Activation to Modulate Cell Death Kinetics.” Cell Death and Differentiation 28: 1644–1657. 10.1038/s41418-020-00691-x. 33335287 PMC8167089

[ptr70389-bib-0047] Qin, Z. , S. Ou , L. Xu , et al. 2021. “Design and Synthesis of Isothiocyanate‐Containing Hybrid Androgen Receptor (AR) Antagonist to Downregulate AR and Induce Ferroptosis in GSH‐Deficient Prostate Cancer Cells.” Chemical Biology & Drug Design 97: 1059–1078. 10.1111/cbdd.13826. 33470049 PMC8168342

[ptr70389-bib-0048] Quaranto, D. , N. R. DeSouza , M. Carnazza , et al. 2025. “Ferroptosis—The “Double‐Edged Sword” in Cancer: Mechanisms of Tumor Suppression/Resistance and Therapeutic Manipulation.” Biology‐Basel 15: 67. 10.3390/biology15010067. 41514908 PMC12784773

[ptr70389-bib-0049] Riegman, M. , L. Sagie , C. Galed , et al. 2020. “Ferroptosis Occurs Through an Osmotic Mechanism and Propagates Independently of Cell Rupture.” Nature Cell Biology 22: 1042–1048. 10.1038/s41556-020-0565-1. 32868903 PMC7644276

[ptr70389-bib-0050] Rodriguez, R. , S. L. Schreiber , and M. Conrad . 2022. “Persister Cancer Cells: Iron Addiction and Vulnerability to Ferroptosis.” Molecular Cell 82: 728–740. 10.1016/j.molcel.2021.12.001. 34965379 PMC9152905

[ptr70389-bib-0051] Shah, R. , M. S. Shchepinov , and D. A. Pratt . 2018. “Resolving the Role of Lipoxygenases in the Initiation and Execution of Ferroptosis.” ACS Central Science 4: 387–396. 10.1021/acscentsci.7b00589. 29632885 PMC5879472

[ptr70389-bib-0052] Shen, W.‐Y. , H. Liang , and Z.‐F. Chen . 2026. “Recent Advances in Metal Complexes and Nanostructured Materials for Enhanced Chemodynamic Therapy.” Dalton Transactions 55: 2418–2427. 10.1039/d5dt02354h. 41527985

[ptr70389-bib-0053] Sundaram, M. K. , R. Preetha , S. Haque , et al. 2022. “Dietary Isothiocyanates Inhibit Cancer Progression by Modulation of Epigenome.” Seminars in Cancer Biology, Epigenetic Regulation of Cancer Progression: Promises and Progress 83: 353–376. 10.1016/j.semcancer.2020.12.021. 33434642

[ptr70389-bib-0054] Trachootham, D. , Y. Zhou , H. Zhang , et al. 2006. “Selective Killing of Oncogenically Transformed Cells Through a ROS‐Mediated Mechanism by Beta‐Phenylethyl Isothiocyanate.” Cancer Cell 10: 241–252. 10.1016/j.ccr.2006.08.009. 16959615

[ptr70389-bib-0055] Wang, Q. , D. Li , L. Liu , Y. Shan , and Y. Bao . 2024. “Dietary Isothiocyanates and Anticancer Agents: Exploring Synergism for Improved Cancer Management.” Frontiers in Nutrition 11: 1386083. 10.3389/fnut.2024.1386083. 38919393 PMC11196812

[ptr70389-bib-0056] Wang, W. , Z. Zhong , S. Peng , et al. 2024. ““All‐In‐One” Metal Polyphenol Network Nanocapsules Integrated Microneedle Patches for Lipophagy Fueled Ferroptosis‐Mediated Multimodal Therapy.” Journal of Controlled Release 373: 599–616. 10.1016/j.jconrel.2024.07.063. 39074587

[ptr70389-bib-0057] Wang, Y. , R. Yu , L. Wu , and G. Yang . 2021. “Hydrogen Sulfide Guards Myoblasts From Ferroptosis by Inhibiting ALOX12 Acetylation.” Cellular Signalling 78: 109870. 10.1016/j.cellsig.2020.109870. 33290842

[ptr70389-bib-0058] Wu, H. , Q. Liu , X. Shan , W. Gao , and Q. Chen . 2023. “ATM Orchestrates Ferritinophagy and Ferroptosis by Phosphorylating NCOA4.” Autophagy 19: 2062–2077. 10.1080/15548627.2023.2170960. 36752571 PMC10283418

[ptr70389-bib-0060] Yang, W. S. , and B. R. Stockwell . 2008. “Synthetic Lethal Screening Identifies Compounds Activating Iron‐Dependent, Nonapoptotic Cell Death in Oncogenic‐RAS‐Harboring Cancer Cells.” Chemistry & Biology 15: 234–245. 10.1016/j.chembiol.2008.02.010. 18355723 PMC2683762

[ptr70389-bib-0061] Yang, W. S. , and B. R. Stockwell . 2016. “Ferroptosis: Death by Lipid Peroxidation.” Trends in Cell Biology 26: 165–176. 10.1016/j.tcb.2015.10.014. 26653790 PMC4764384

[ptr70389-bib-0062] Ye, L. , Y. Qiao , P. Wang , et al. 2025. “Nanoengineered Hydrogels Disrupt Tumor Antioxidant Defense via Photothermal‐Chemodynamic Synergy and Oxidative Stress Boosts.” Journal of Nanobiotechnology 23: 584. 10.1186/s12951-025-03626-1. 40849478 PMC12374360

[ptr70389-bib-0063] Zhang, C. , X. Liu , S. Jin , Y. Chen , and R. Guo . 2022. “Ferroptosis in Cancer Therapy: A Novel Approach to Reversing Drug Resistance.” Molecular Cancer 21: 47. 10.1186/s12943-022-01530-y. 35151318 PMC8840702

[ptr70389-bib-0064] Zhang, S. , W. Xin , G. J. Anderson , et al. 2022. “Double‐Edge Sword Roles of Iron in Driving Energy Production Versus Instigating Ferroptosis.” Cell Death & Disease 13: 40. 10.1038/s41419-021-04490-1. 35013137 PMC8748693

[ptr70389-bib-0065] Zhen, C. , J. Li , J. Liu , Y. Lyu , L. Xie , and H. Lv . 2023. “Phenethyl Isothiocyanate Induces Oxidative Cell Death in Osteosarcoma Cells With Regulation on Mitochondrial Network, Function and Metabolism.” Biochimica et Biophysica Acta ‐ Molecular Basis of Disease 1869: 166740. 10.1016/j.bbadis.2023.166740. 37142133

[ptr70389-bib-0066] Zheng, K. , Y. Dong , R. Yang , Y. Liang , H. Wu , and Z. He . 2021. “Regulation of Ferroptosis by Bioactive Phytochemicals: Implications for Medical Nutritional Therapy.” Pharmacological Research 168: 105580. 10.1016/j.phrs.2021.105580. 33781874

[ptr70389-bib-0067] Zhou, Q. , Y. Meng , D. Li , et al. 2024. “Ferroptosis in Cancer: From Molecular Mechanisms to Therapeutic Strategies.” Signal Transduction and Targeted Therapy 9: 55. 10.1038/s41392-024-01769-5. 38453898 PMC10920854

[ptr70389-bib-0068] Zhu, H. , C. Yu Chan , J. Z. Xiong Heng , et al. 2025. “Bioactive Metal Sulfide Nanomaterials as Photo‐Enhanced Chemodynamic Nanoreactors for Tumor Therapy.” Nanoscale Horizons 10: 1307–1329. 10.1039/D5NH00122F. 40293306

[ptr70389-bib-0069] Zou, Q. , X. Zhou , J. Lai , et al. 2025. “Targeting p62 by Sulforaphane Promotes Autolysosomal Degradation of SLC7A11, Inducing Ferroptosis for Osteosarcoma Treatment.” Redox Biology 79: 103460. 10.1016/j.redox.2024.103460. 39657365 PMC11681892

